# Interplay between obesity-associated insulin resistance and immune system through the lens of evolutionary medicine

**DOI:** 10.1016/j.molmet.2026.102335

**Published:** 2026-02-13

**Authors:** Maryam Moazzam-Jazi, Saeideh Jafarinejad-Farsangi, Leila Najd-Hassan-Bonab, Maryam Daneshpour, Zhaoli Liu, Manoj Kumar Gupta, Ramakrishna Vadde

**Affiliations:** 1 Cellular and Molecular Endocrine Research Center, Research Institute for Endocrine Molecular Biology, Research Institute for Endocrine Sciences, Shahid Beheshti University of Medical Sciences, Tehran, Iran; 2 Physiology Research Center, Institute of Neuropharmacology, Kerman University of Medical Sciences, Kerman, Iran; 3 Central China Subcenter of National Center for Cardiovascular Diseases, Henan Cardiovascular Disease Center, Fuwai Central-China Cardiovascular Hospital, Central China Fuwai Hospital of Zhengzhou University, Zhengzhou, 450046, China; 4 Hematology, Hemostasis, Oncology, and Stem Cell Transplantation, Hannover Medical School, Hannover, 30625, Germany; 5 Department of Biotechnology and Bioinformatics, Yogi Vemana University, Kadapa, 516005, Andhra Pradesh, India

**Keywords:** Allergy, Evolutionary medicine, Insulin resistance, Inflammation, Purifying selection, *PTEN*

## Abstract

Insulin resistance (IR), commonly associated with obesity, is linked to a range of metabolic and immune-related disorders in the contemporary human population. Nevertheless, it is evolutionary well-conserved, suggesting its potential survival advantages to our ancestors. This review aims to explore the intricate interplay between IR and the immune system as well as its implications for the development of immune-metabolic and allergic diseases in the modern era. From an evolutionary medicine perspective, the longevity of ancient humans relied on energy storage to endure food shortages and effectively activate the immune system against various diseases. Under normal conditions, insulin induces glycogen and triglyceride synthesis in the liver and adipose tissues. However, IR directs more glucose to insulin-independent tissues, such as the immune system, which are critical for survival in adverse conditions. The persistent IR in our current lifestyle promotes low-grade inflammation, accompanied by various metabolic and allergic disorders. Critically, this evolutionary mismatch not only explains disease susceptibility but also informs therapeutic design to target immune-metabolic crosstalk. Moreover, our evolutionary analysis demonstrates that the genomic regions near the *PTEN, IL27,* and *NUPR1* genes could play an important role in this interaction across diverse populations.

## 1 Introduction

Obesity, defined as a body mass index (BMI) of 30 kg/m^2^ or higher [1,2], is a complex, multifactorial condition that may detrimentally affect health [3,4]. Over the past few decades, obesity rates have increased dramatically, affecting populations across various age groups and ethnicities [5]. Obesity-associated persistent chronic inflammation disrupts insulin signaling, causing insulin resistance (IR), which contributes not only to type 2 diabetes (T2D) [6], but also to various immune system-mediated disorders, such rheumatoid arthritis (RA), inflammatory bowel disease (IBD) [6], and multiple sclerosis [7]. This association was also observed during the recent pandemic, where individuals with obesity were at higher risk of developing severe COVID-19 infection [8]. The close relationship between IR and the immune dysfunction is further supported by the fact that individuals with autoimmune diseases, such as IBD and RA, have a higher chance of developing IR [9]. These autoimmune features not only complicate treatment, for example, by reducing the efficacy of glucocorticoid therapy, but also underscore the role of chronic inflammation in driving IR. Additionally, obesity and IR are associated with increased asthma risk, greater disease severity, and reduced response to standard bronchodilator and corticosteroid therapies [10]. According to experimental evidence, insulin-lowering medications, particularly metformin, can alleviate the hyperinsulinemia and airway inflammation in these patients, improving asthma outcomes [[11], [12], [13]]. Thus, it is time to reconsider the separation of IR and immune-related diseases, advocating for a perspective that views this condition through an immune-etiopathogenic lens. Reframing these conditions in this way may open avenues for integrated therapeutic strategies targeting inflammation and addressing the root causes of these interconnected disorders. In this context, recently developed evolutionary medicine (EM) might be helpful, as it examines how past evolutionary processes have influenced human vulnerability to diseases by considering factors such as genetics, behavior, environment, and microbes. Unlike conventional medicine's focus on immediate causes, EM explores the evolutionary reasons behind certain traits and diseases, offering a more comprehensive understanding of health challenges [[14], [15], [16], [17]]. By integrating evolutionary perspectives with conventional medical knowledge, EM approaches may provide a more complete picture of why we get sick and how to prevent and treat diseases in the contemporary human population [18,19].

From an evolutionary medicine perspective, insulin resistance is a conserved physiological response that occurs during acute conditions such as starvation, infection, inflammation, and physical or psychological stress, reflecting adaptive energy reallocation rather than primary pathology [20,21]. Under normal conditions, energy storage, primarily as hepatic glycogen and adipose triglycerides, is regulated by insulin. During acute stress, infection, or trauma, pro-inflammatory cytokines and stress hormones temporarily reduce insulin sensitivity in peripheral tissues such as muscle and fat. This limits glucose uptake in these tissues, preserving glucose for immune-related biosynthetic processes essential for host defense. Meanwhile, energy demands are met mainly through the oxidation of fatty acids and ketone bodies, allowing immune cells and stress-response systems to operate under limited glucose availability. This adaptive response is further supported by the fructose survival hypothesis [22], which proposes that fructose metabolism activates a short-term survival program characterized by insulin resistance, fat accumulation, and reduced mitochondrial oxidative phosphorylation. Although this response is intended to be modest and short-lived, its chronic activation in contemporary environments, driven by fructose-rich Western diets and persistent caloric excess, results in persistent insulin resistance, obesity, metabolic syndrome, and type 2 diabetes. Thus, insulin resistance likely evolved as a transient metabolic adaptation that supported immune function and survival during periods of acute stress; however, its constant activation in modern environments promotes chronic metabolic and inflammatory diseases.

This review explores the interplay between insulin resistance and the immune system throughout human evolution, expanding the evolutionary lens to include immune features beyond classical metabolic stress, such as IgE-mediated allergic responses or immune training disruptions in modern environments. We examined evolutionary trends in genomic regions influencing immunity and insulin resistance across populations. This integrative understanding not only offers promising avenues for novel therapeutic and preventive strategies targeting obesity-related disorders but also underscores the critical role of insulin resistance in immune-related diseases, highlighting its broader significance in human health and disease.

## 2 Overview of the insulin signaling pathway

**Figure 1:**
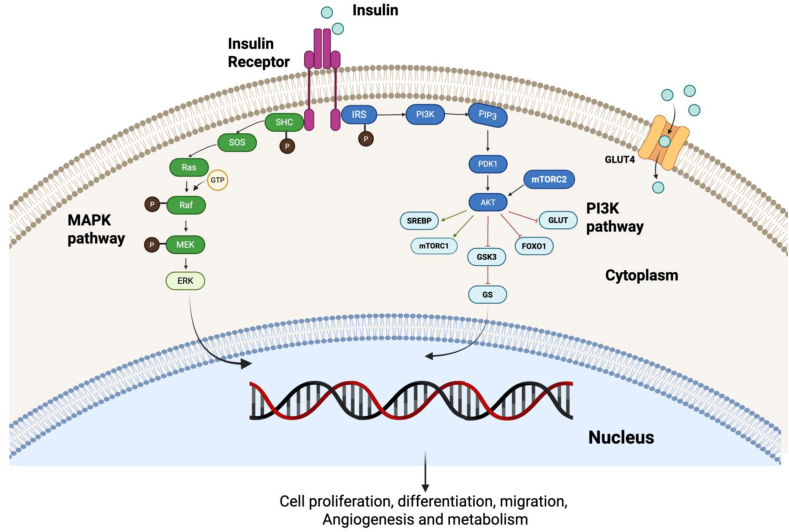
**Insulin signaling pathway.** After binding the insulin to its receptor, a conformational change and autophosphorylation of the receptors lead to the recruitment and phosphorylation of receptor substrates such as IRS and SHC proteins, and trigger the signaling pathways. PI3K/AKT and MAPK pathways are activated in parallel in healthy conditions, but insulin resistance disrupts the PI3K/AKT.

The insulin signaling pathway is initiated when insulin binds to its transmembrane receptor, inducing its autophosphorylation. This activated receptor then phosphorylates tyrosine residues on key substrate proteins, primarily the Insulin Receptor Substrate (IRS) and Src homology 2 domain-containing (SHC) protein (Fig. 1). As a result, two key pathways, including the Ras/mitogen-activated protein kinase (MAPK) and the phosphoinositide 3-kinase (PI3K)/AKT pathways, are activated [23,24]. Both MAPK and PI3Ks are evolutionarily conserved signaling pathways that regulate diverse physiological processes [[25], [26], [27]]. In the MAPK branch of the insulin signaling pathway, insulin binds to its receptor, leading to the recruitment and phosphorylation of the adaptor protein SHC. Subsequently, the son-of-sevenless (SOS) protein, a guanine nucleotide exchange factor (GEF), facilitates the shift of inactive membrane-bound Ras (GDP-Ras) to an active GTP Ras. The activated Ras interacts with downstream components, including serine–threonine kinase Raf, which phosphorylates and activates MEK and ERK. The activated *ERK* translocates to the nucleus, regulating the expression of different genes involved in diverse processes such as cell proliferation, differentiation, and survival [28,29].

In the PI3K/AKT pathway, AKT is phosphorylated via phosphoinositide-dependent protein kinase-1 (PDK1) at the kinase domain and mammalian target of rapamycin complex 2 (mTORC2) at the carboxy-terminal regulatory domain to modulate glucose and lipid metabolism, resulting in regulating the translocation of glucose transporters (GLUT), glycogen synthase kinase 3 (GSK3), forkhead box O1 transcription factor (FoxO1), and mammalian Target of Rapamycin Complex 1 (mTORC1). Similarly, the activated PI3K/AKT pathway regulates lipid metabolism by promoting cholesterol and fatty acid accumulation via sterol regulatory element-binding proteins (SREBP) and inhibiting lipolysis [23,24]. This pathway is tightly regulated by lipid phosphatases such as PTEN and SHIP2, which act as negative modulators by dephosphorylating PI (3,4,5) P3, a key second messenger in insulin signaling. PTEN, in particular, has garnered significant attention not only for its role in metabolic regulation but also as a tumor suppressor frequently mutated in human cancers. It influences multiple cellular processes, including survival, proliferation, migration, and invasion, partly through dephosphorylation of protein substrates like focal adhesion kinase (FAK). In metabolic tissues such as skeletal muscle and adipose tissue, increased PTEN or SHIP2 activity impairs insulin signaling and promotes insulin resistance, whereas decreased activity of these phosphatases enhances insulin sensitivity [30]. Under normal physiologic conditions, both the PI3K/AKT and MAPK pathways work in parallel via insulin to regulate cell proliferation, differentiation, migration, angiogenesis, and metabolism [31,32]. However, disruption of the PI3K/AKT pathway causes insulin resistance [33]. In response, the pancreas increases insulin production to counter the diminished cellular response and lower blood sugar. However, prolonged hyperinsulinemia weakens signaling pathways such as PI3K/AKT, hindering the movement of glucose transporter protein (GLUT 4) to the cell surface. As a result, glucose uptake becomes less effective, contributing to sustained high blood glucose levels [23,34]. Also, the metabolic and physiological characteristics of tissues can determine the impact of insulin resistance. For instance, adipose tissue plays a crucial role in regulating overall insulin sensitivity and energy balance in the body [35]. On the one hand, insulin signaling in adipocytes suppresses lipolysis by reducing cAMP production via the PI3K/AKT pathway and inhibiting FoxO1-driven transcription of lipolytic enzymes like ATGL. At the same time, insulin promotes triacylglycerol synthesis by increasing GLUT4-mediated glucose uptake, which provides glycerol 3-phosphate for fat esterification [36]. However, it also limits glycerol 3-phosphate production from glucose to prevent excessive fat buildup [37]. Disruption of this balance, impaired lipolysis, and increased lipogenesis can lead to TAG accumulation and obesity.

## 3 Metabolic regulation in leukocytes: resting vs. activated states

**Figure 2:**
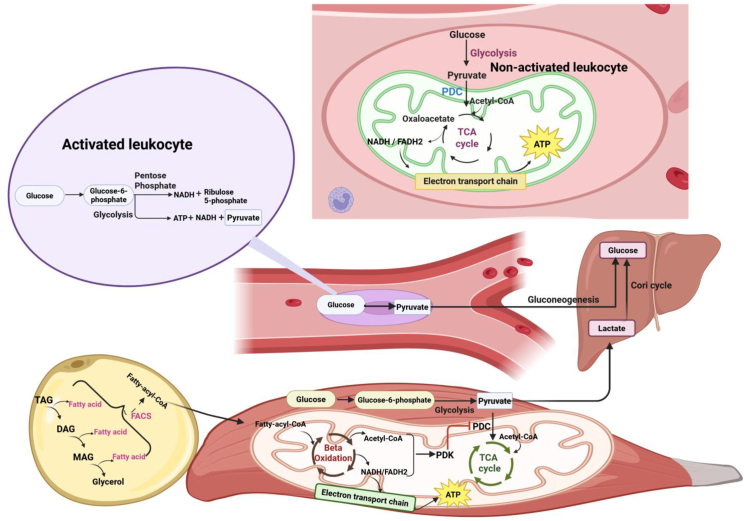
**Metabolic regulation in leukocytes in resting and activated states.** The non-activated (resting) leukocytes primarily use oxidative phosphorylation to produce ATP from glucose through the full TCA cycle. However, activated leukocytes shift the complete glucose metabolism to partial oxidation to meet the much higher energy demands required under unfavorable conditions. The partial oxidation of glucose occurs through glycolysis and the PP pathway. At the same time, TAGs' lipolysis and oxidation in adipocytes release fatty acids to the circulation as an alternative energy source for skeletal muscle, liver, and the immune system. Acetyl-CoA and nicotinamide adenine dinucleotide (NADH), through fatty acid oxidation, can allosterically activate pyruvate dehydrogenase kinase (PDK), which inhibits the pyruvate dehydrogenase complex (PDC), leading to partial oxidation of glucose through the TCA cycle. Hence, the partially oxidized glucose in the peripheral tissues is converted to pyruvate and lactate, which reaches the liver and is converted back to glucose via the Cori cycle.

Glucose metabolism in leukocytes is dynamically regulated to meet their functional and energetic demands during immune system activation. In the resting (non-activated) leukocytes, energy is mainly generated through mitochondrial oxidative phosphorylation (OXPHOS); glycolysis produces pyruvate, which is converted to acetyl-CoA and oxidized in the tricarboxylic acid (TCA) cycle, generating NADH/FADH_2_ that fuel the electron transport chain and ATP synthesis [38]. Upon immune activation by factors such as trauma, infection, or disease, leukocytes often shift away from complete oxidation of glucose toward aerobic glycolysis (the Warburg effect), in which more glucose is converted to pyruvate and then lactate despite oxygen availability (Fig. 2). In parallel, glucose flux through the pentose phosphate pathway (PPP) increases, supporting NADPH and biosynthetic intermediates production together with enhanced glycolysis and rapid ATP generation [20].

This metabolic adaptation occurs alongside, and may be supported by inflammation-induced insulin resistance in peripheral tissues, which helps maintain circulating glucose availability for the immune cells. Meanwhile, lipolysis of triacylglycerols (TAGs) and oxidation in adipocytes release free fatty acids into the circulation, serving as an alternative energy source for skeletal muscle, liver, and the immune cells (e.g., regulatory T cells, M2 macrophages, and memory lymphocytes). Fatty acids can produce higher ATP than glucose, highlighting them as the primary substitute energy for humans and most animals under certain conditions. Additionally, the generation of acetyl-CoA and nicotinamide adenine dinucleotide (NADH) via fatty acid oxidation can allosterically activate pyruvate dehydrogenase kinase (PDK) and inhibit the pyruvate dehydrogenase complex (PDC), thereby limiting pyruvate entry into the TCA cycle and shifting glucose metabolism toward lactate production rather than complete mitochondrial oxidation Hence, in peripheral tissues a greater fraction of glucose is converted to pyruvate and subsequently to lactate that is transported to the liver and converted back to glucose via the Cori cycle (Fig. 2) [39,40]. This integrated reprogramming ensures metabolic flexibility during stress, even at the expense of temporarily inducing insulin resistance.

From an evolutionary perspective, immune activation does not rely on a single metabolic program; instead, distinct immune cell subsets exhibit specialized metabolic rewiring that reflects adaptive division of labor and resource-allocation trade-offs, potentially shaped by natural selection. Proinflammatory M1 macrophages exhibit high glucose uptake and lactate production, relying mainly on aerobic glycolysis, whereas anti-inflammatory M2 macrophages depend primarily on oxidative phosphorylation (OXPHOS). Evidence suggests that OXPHOS, rather than glycolysis, is the key metabolic pathway supporting M2 polarization, as inhibiting glycolysis does not significantly impair M2 differentiation and can even block M1 polarization in M2 macrophages. In addition to OXPHOS, fatty acid oxidation (FAO) is often associated with the M2 phenotype and is promoted by peroxisome proliferator–activated receptor (PPAR) signaling, which supports M2 polarization. However, FAO's role is context-dependent: its inhibition does not always disrupt M2 polarization and can also promote inflammasome activation in M1 macrophages, underscoring the complexity of macrophage metabolic regulation [41]. T-cell metabolism varies with phenotype and functional state [41]. Naive T cells are metabolically quiescent with minimal energy demands. Upon differentiation into effector T cells, metabolic activity markedly increases to support rapid proliferation and biomass synthesis, leading to active metabolism. In contrast, regulatory T (Treg) cells exhibit distinct immunosuppressive metabolic characteristics relative to both naive and effector T cells. Memory T cells also possess unique metabolic features that differentiate them from other T-cell subsets. Although enhanced OXPHOS is a shared metabolic feature of memory T (Tm) cells, distinct Tm subsets exhibit differences in their reliance on OXPHOS versus glycolysis and in substrate utilization [42]. Effector memory T (Tem) cells depend less on OXPHOS than central memory (Tcm) or tissue-resident memory (Trm) cells. Memory T cells can still develop when glycolysis is enforced through deletion of von Hippel–Lindau (VHL), but this condition preferentially drives a Tem phenotype. While Tcm and Trm cells show similar OXPHOS dependence, they differ metabolically in substrate use, with Trm cells uniquely relying on exogenous fatty acids to sustain mitochondrial respiration.

Consistent with life history theory, which recognizes heterogeneity and trade-offs in immune-metabolic programs under adverse conditions (e.g., infection, injury, and limited energy availability) [43], we extend this framework by proposing that these established immune-subset–specific metabolic programs function as a coordinated system that is supported at the organismal level by transient, inflammation-associated insulin resistance. The glycolytic programs in effector immune cells prioritize rapid pathogen control [44,45], whereas oxidative and FAO-dependent programs in regulatory and memory cell populations favor tissue maintenance and immune recall [46,47]. At the organismal level, the transient inflammation-associated insulin resistance may have evolved as a systemic energy-allocation strategy that supports these parallel, task-specific immune programs by preserving circulating glucose for high-demand effector responses while directing lipid substrates to tissues and immune subsets optimized for oxidative metabolism. However, in modern obesogenic and inflammatory environments, chronic activation of this response contributes to persistent insulin resistance and metabolic diseases.

## 4 Obesity-associated insulin resistance and immune dysfunction

During obesity, both innate and adaptive immune cells experience systemic pro-inflammatory polarization, increasing the secretion of pro-inflammatory cytokines. This, in turn, leads to low-grade inflammation and insulin resistance [48].

### 4.1 Innate immune dysregulation in obesity

Innate immune cells, while essential for acute defense, drive chronic pathology when persistently activated by obesogenic signals. Elevated circulating monocytes in individuals with obesity are recruited to adipose tissue and differentiate into macrophages, a process modulated by fasting insulin levels and dietary patterns. Diet more similar to our ancestral patterns, such as the Mediterranean diet, are associated with less inflammatory monocyte profiles [49], a lower risk of chronic diseases, and greater longevity compared to the Western diet [[50], [51], [52], [53]]. This supports the concept that modern nutritional environments are mismatched with evolved immune-metabolic regulation, promoting chronic inflammation, obesity, and cardiovascular disease. Once recruited, these macrophages disrupt the homeostatic balance in adipose tissue. In lean individuals, macrophages primarily adopt an anti-inflammatory M2 phenotype that supports tissue repair and insulin sensitivity. However, obesity drives a shift toward pro-inflammatory M1 macrophages, which create a low-grade chronic inflammation milieu. Simultaneously, this systemic immune dysregulation exacerbates type 2 inflammatory pathways in distant tissues such as respiratory system, linking obesity to increased susceptibility to asthma and allergic airway inflammation. Similarly, early-life exposure to allergens and viruses results in metabolic reprogramming and immune training, which is correlated with augmented allergen-related airway inflammation and elevated risk of allergic diseases [54].

Beyond adipose tissue, this dysregulation affects other innate populations, such as eosinophils. Evolutionarily, elevated eosinophil activity might have been advantageous in ancestral environments characterized by high parasitic burdens, [55,56]. In these settings, eosinophil-mediated type 2 immune responses provided a dual survival benefit by promoting the expulsion of parasitic helminths and inducing tissue tolerance to repair the physical damage caused by parasites and maintain organ function [57]. Furthermore, adipose tissue eosinophils likely evolved to provide metabolic homeostasis during these energetic stresses by secreting IL-4 to sustain anti-inflammatory M2 macrophages and promote safe lipid storage [58]. In such situations, immune–metabolic balance was maintained through modulated, short-term insulin resistance, which allowed optimized energy redistribution to energize immune functions without the detrimental effects observed in chronic insulin resistance. In contrast, today's persistent low-grade inflammatory state results in sustained insulin resistance and is accompanied by reduced eosinophil abundance, suggesting a shift from adaptive immune-metabolic flexibility to maladaptive metabolic diseases. This maladaptation is additionally driven by Toll-like receptors (TLRs), which evolved to detect pathogen-associated molecular patterns (PAMPs) and tissue damage-associated molecular patterns (DAMPs). Saturated fatty acids derived from nutrient excess bind to TLR2 and TLR4 on macrophages, activating inflammatory signaling pathways (e.g., NF-κB) and producing pro-inflammatory cytokines (e.g., IL-6). This signaling induces metabolic reprogramming (such as increased glycolysis and mTORC1 activity), leading to a persistent “trained immunity” state that worsens local insulin resistance [59,60]. Similarly, the binding of TLRs to environmental lipopolysaccharides (LPS) or allergens in airway epithelial cells triggers inflammatory pathways that favor asthma development. Both scenarios involve a shared intracellular signaling pathway, primarily through the MyD88-dependent pathway, which leads to NF-κB and MAPK activation, subsequent pro-inflammatory cytokine production, and immune response [61,62]. This convergence illustrates how diverse environmental signals (e.g., dietary or allergenic) exploit the same conserved signaling architecture to drive metabolic and inflammatory diseases [54,60].

### 4.2 Adaptive immune remodeling in obesity

The adaptive immune system evolved to provide precise, long-term protection against specific pathogens while maintaining tolerance to self-antigens. However, our modern environment can act as a chronic stressor that disrupts this evolutionary calibration. Obesity may impair humoral immunity (mediated by B-lymphocytes), impacting infection susceptibility and vaccine efficacy [63]. For example, while individuals with obesity initially produce typical levels of influenza-specific IgM and IgG following vaccination, these titers decline more rapidly than in individuals without obesity [64]. This accelerated decay likely shows an energetic trade-off, where chronic inflammation exhausts the metabolic resources required to maintain long-lived plasma cells, rendering individuals with obesity more susceptible to infection over time. Moreover, T cell remodeling drives inflammation in adipose tissue. Both cytotoxic CD8+ and CD4+ T cells generally increase during obesity [65]. Specifically, there is an increase in CD4+ T cell infiltration into visceral adipose tissue (VAT), where these cells adopt an active Th1 phenotype with higher IFN-γ production, contributing to adipose tissue inflammation and metabolic dysfunction. Conversely, Th2 cells and regulatory T cells (Tregs) are depleted in obese VAT. Their presence negatively correlates with systemic inflammation and insulin resistance, exerting protective effects. Adipose tissue macrophages (ATMs) use MHC II to present obesity-related antigens to T cells, mis-interpreting metabolic stress as a pathogenic threat. Crucially, MHC II deficiency reduces CD4+ T cell accumulation and inflammation in VAT, highlighting the importance of antigen presentation by ATMs in obesity-induced adipose inflammation and insulin resistance [[66], [67], [68], [69], [70], [71]].

This failure of immune tolerance extends to allergic disease. Treg cell dysfunction or reduced frequency is involved in the development of autoimmune and allergic diseases/reactions, possibly through elevated IgE levels [72]. The binding of allergen-specific IgE antibodies to their high-affinity receptor (FcεRI) on mast cells and basophils triggers the release of inflammatory molecules such as histamine, which cause inflammation and allergic symptoms [73]. While immunotherapy with Omalizumab (anti-IgE) alleviates airway inflammation by improving Treg cell homeostasis in children with severe asthma [74], obesity adversely affects drug response and disease management [75]. Therefore, obesity represents a fundamental mismatch between our evolved immune architecture and our current (nutritional) environment, transforming adaptive flexibility into chronic pathology.

## 5 Insulin resistance and the immune system: an evolutionary insight

Insulin resistance has been conserved through evolution [21,39]. Insulin resistance plays a crucial role in conserving glucose for essential functions during periods of starvation, immune activation, and cellular biosynthesis, redirecting glucose from oxidation in the TCA cycle and instead relying on fatty acids and ketone bodies for providing energy. While historically adaptive, allowing humans to survive under nutrient scarcity and metabolic stress, chronic IR in modern environments now predisposes individuals to health issues such as type 2 diabetes and cardiovascular disease [20].

### 5.1 Insulin resistance as an evolutionary maladaptation in contemporary humans

In 1962, James V. Neel hypothesized that IR might have initially been an adaptive trait, which later became problematic due to changes in lifestyle and diet [76]. In brief, Neel's thrifty genotype hypothesis proposes that specific genes, known as thrifty genes linked to energy storage, might have been selected in populations that historically faced periods of food scarcity. These genes enabled effective energy utilization during times of plenty, promoting survival. However, in contemporary environments with consistent access to food, they might have led to the development of metabolic disorders such as obesity and type 2 diabetes. Sometimes, the thrifty genotype has also been used as an umbrella concept for genes thought to be favored in certain environments [17]. Although some studies suggest links between genetic variants and ecological or climatic stress, the idea remains controversial. Notably, despite widespread adoption of Western lifestyles, only about 20–30% of people develop obesity, and there is limited evidence that ancestral populations experienced the frequent, severe famines needed to select for thrifty genes [77,78].

Additionally, Neel's thrifty genotype hypothesis has been criticized by several researchers because it is well-documented that IR and its associated complications are polygenic [17,[79], [80], [81], [82], [83], [84], [85]]. Several genetic variants with minor to modest effect sizes are involved in IR incidence [[79], [80], [81]]. Therefore, it is less likely that selection has acted to preserve every variant with negligible effect. Several independent studies have also failed to support the idea that genetic variants associated with an increased risk of obesity and type 2 diabetes were positively selected, contradicting the thrifty genotype hypothesis [[86], [87], [88]]. In 2008, the drifty gene hypothesis, proposed by Speakman, emerged as a counterpoint to the traditional thrifty gene hypothesis. It contends that the prevalence of thrifty (energy storage) genes is not due to positive selection, but genetic drift resulting from removing predatory selection pressure [85]. Based on this theory, for the first time in evolutionary history, around 2 million years ago, our ancient ancestors, *Homo habilis* and *Homo erectus*, acquired the capability to escape from predators. Therefore, essential genes for energy storage drifted during human evolution rather than being removed by natural selection [85,89]. This hypothesis is closely aligned with the dual intervention point model, which suggests that body energy reserves are regulated by two physiological thresholds: a lower limit, below which the risk of starvation activates strong biological responses to restore energy balance, and an upper limit, beyond which the historical threat of predation would have triggered mechanisms to reduce excess energy stores. As early humans faced fewer predators due to technological and social advancements, the upper limit became less relevant. This relaxation of selective pressure may have allowed genetic drift to increase individual variation in the tendency to accumulate energy reserves. In modern environments with abundant food availability, this results in a greater predisposition to excess weight in some individuals, not because energy storage was ever advantageous, but because evolutionary constraints on maximum energy accumulation were no longer enforced [85].

Although both the thrifty and drifty hypotheses have pointed to the possible genetic susceptibility to insulin resistance, other researchers suggested that neither theory can fully account for the current IR prevalence and its subsequent outcomes worldwide. In 1992, Hales and Barker proposed another critical theory, namely the thrifty phenotype hypothesis. It states that poor intrauterine nutrition during fetal development can lead to decreased body size of fetuses as an adaptation strategy to an inadequate energy supply. This results in giving birth to an underweight infant, who has a greater chance of developing IR and obesity compared to normal-weight infants [74]. However, environmental alterations during the early stages of an organism's development can make the selected trajectory inappropriate, resulting in negative impacts on health later. A recent study suggested that the phosphatase and tensin homolog (*PTEN*) gene may act as a thrifty gene, which historically helped store energy to survive food scarcity but has become harmful in today's obesogenic environment. During prenatal development, nutrient availability influences *PTEN* activity through epigenetic methylation. Specifically, deficiency of dietary protein, during pregnancy, and choline increases the activity of DNA methyltransferases (DNMTs), particularly DNMT1 and DNMT3a, which methylate the promoter region of the *PTEN* gene, suppressing its expression [90]. The reduced expression of *PTEN* as a negative regulator of the insulin signaling pathway (PI3K/AKT), results in increased insulin sensitivity, affecting both glucose homeostasis and cell survival [[91], [92], [93]]. However, in environments with abundant food and sedentary lifestyles, this increased insulin sensitivity can promote excessive fat accumulation and cell proliferation, raising the risk of obesity, metabolic disorders, and cancer. For example, individuals with *PTEN* mutations are more insulin sensitive but also more prone to obesity and certain cancers. Thus, while increased insulin sensitivity may seem beneficial, it predisposes to diseases associated with energy surplus in calorie-rich settings [92,93].

Besides, Wang proposed the adjustable threshold hypothesis to explain insulin resistance [94]. According to this theory, the insulin signaling pathway could operate as a bistable system in which a cell responds to insulin as all or none at two threshold insulin concentrations. It appears that the thresholds are adjustable to adapt to extreme conditions. For instance, during pregnancy, the thresholds constantly elevate to boost the mother's insulin resistance and provide the increasing glucose demand for the expanding fetal brain. Hence, based on this theory, insulin is necessary to preserve glucose in the brain. The cell response to the insulin signal switches from none to all as the insulin concentration is enhanced. Similarly, the cell response switches from all to none, along with decreasing the insulin concentration; different switching points reminisce the hysteresis, which is one of the key components of the threshold adjustable hypothesis. Here, hysteresis can be quantified through the difference between the two thresholds. This hypothesis highlights that the delayed switch-on of insulin action results in spare glucose for the brain, while the lagged switch-off prevents hyperglycemia; therefore, the action of insulin essentially serves as a restricting, rather than a stimulating, signal for glucose uptake. Thus, understanding the evolutionary metabolic mismatch can facilitate the development of medications for contemporary metabolic and immune disorders, which are discussed in the next sections.

### 5.2 Insulin resistance as a defense mechanism

Infectious pathogens have been strong selective forces during human evolution. Over 100,000 years, migrations and cultural changes exposed people to new diseases, denser populations, and vectors [95]. Recent discoveries highlight an endocrine–immune axis in which insulin plays a critical role in regulating immune cell functions and metabolic processes, thereby influencing immune responses to infection. Emerging evidence reveals that insulin is not merely a metabolic hormone but a key modulator of immune cell function [96]. For instance, in 2014, Straub proposed IR as a catabolic process that was positively selected during human evolution to provide sufficient glucose (energy) for the selfish immune system and the brain, thereby ensuring the activation of either defense or mental systems. Based on this hypothesis, energy expenditure and storage are important factors exposed to selection during evolution. In demanding energy situations, various biological pathways, including IR, are positively selected for short-term benefits, while they are negatively selected in chronic situations [97].

In this context, the fructose survival hypothesis [22] proposes that obesity and metabolic disorders result from chronic overactivation of an evolutionarily conserved survival program that originally evolved to enhance fitness during periods of anticipated stress. This response, triggered by dietary fructose intake or endogenous fructose production via the polyol pathway, promotes hunger, fat accumulation, insulin resistance, systemic inflammation, and increased blood pressure. Unlike other nutrients, fructose depletes intracellular ATP and impairs its regeneration through mechanisms involving uric acid accumulation, mitochondrial oxidative stress, inhibition of AMP-activated protein kinase, and stimulation of vasopressin signaling, leading to suppressed mitochondrial oxidative phosphorylation and enhanced glycolysis. While this response is intended to be transient, its persistent activation in humans, driven by thrifty genetic backgrounds and fructose-rich Western diets, contributes to obesity and a broad spectrum of metabolic and age-related diseases. This evolutionary logic also extends to allergic immunity. IgE antibody has evolved through a gene duplication event in an ancestral antibody of IgY about 200–300 million years ago in early mammals. Simultaneously, the IgE-specific high-affinity receptors co-evolved on immune cells such as mast cells and basophils [98]. Wild animals show much higher IgE levels than domestic animals and humans, indicating IgE's survival role in pathogen-rich environments. However, in today's hygienic settings, this response has become maladaptive, leading to more allergic diseases due to exaggerated IgE activity [98,99]. A recent study demonstrated that the optimized mast cells activation in response to IgE receptor crosslinking highly depends on glucose uptake through GLUT1 and GLUT3, the insulin-independent glucose transporters [100], effectively linking acute immune defense to glucose prioritization during insulin resistance. Therefore, IR may not only a metabolic defect, but a coordinated immune-metabolic adaptation.

The modern mismatch was also observed during COVID-19 pandemic, where the acute COVID-19 infection frequently induces new-onset insulin resistance, driven by inflammation and immune activation [101,102]. This observation aligns with the acute IR program hypothesis, suggesting that IR evolved as a beneficial response to immediate energy demands during infections. However, this response can lead to maladaptive outcomes in the modern environment, characterized by reduced microbial exposure and chronic inflammatory states. This paradox can be explained by the fact that chronic IR and associated metabolic dysfunction lead to persistent inflammation, impaired immune responses, and increased ACE2 expression in adipose tissue, which together create a more favorable environment for SARS-CoV-2 infection and amplify disease severity [103]. Recent research reveals that modern humans inherited about 2% of their genome, including immune-related genes, from Neanderthals. A haplotype on chromosome 3 (positions 45,859,651–45,909,024, hg19) from Vindija Neanderthals is linked to severe COVID-19, especially common in South Asians and Bangladeshi populations [104]. Possibly once protective against ancient pathogens, but now increasing the risk of severe COVID-19, a vivid example of evolutionary mismatch.

### 5.3 Insulin resistance as a potential consequence of disrupted immune training in the modern context

The hygiene hypothesis proposed by David Strachan [105] may provide a complementary perspective to our discussion. According to this hypothesis, improved early-life hygienic conditions reduce infection risk but impair proper immune system development, potentially leading to chronic inflammatory situations. This may cause an imbalance between Th1 and Th2 cells, with elevated Th2 activity and increased levels of cytokines such as IL-4 and IL-13. Reduced microbial exposure also disrupts the development and function of regulatory T cells, which normally help control excessive immune responses. Malfunctioning Tregs result in impaired regulation of both Th1 and Th2 pathways, increasing the risk of Th2-driven allergic diseases as well as Th1-associated autoimmune conditions [106]. To avoid misleading the public into believing that hygiene is inherently harmful, some researchers have refined this by suggesting that alternative concepts, such as the microbial hypothesis and the old friend's hypothesis, emphasize the importance of specific microbial exposures rather than hygiene per se [107]. This hypothesis proposes that the human immune system co-evolved to depend on regular contact with certain harmless or beneficial organisms, known as “old friends,” which help train and regulate immune responses. From an evolutionary perspective, the host (human ancestors) effectively outsourced key regulatory functions to these symbionts to conserve metabolic energy, relying on their presence to calibrate the threshold for inflammation. In contrast, modern lifestyles, urbanization, and medical practices have greatly reduced exposure to these beneficial organisms. As a result, immune regulation is weakened, increasing susceptibility to inflammatory and immune-related disorders. Exposure to natural environments may help restore some of these regulatory signals, supporting healthier immune function and reducing inflammation [108].

Although several mechanisms have been proposed, the initial triggers of adipose tissue inflammation in obesity remain incompletely understood, particularly regarding microbial contributions. The classical view of adipocyte dysfunction largely assumes that this inflammation is “sterile” and does not consider the possible role of microbes or their toxins. The microbial hypothesis suggests that inflammation may instead be driven by both short-term and long-term factors. Repeated viral infections may trigger temporary inflammation, which can become chronic when combined with persistent conditions such as gut dysbiosis and metabolic endotoxaemia, in which bacterial toxins, such as lipopolysaccharide, enter the bloodstream. This continuous exposure may explain the low-grade, chronic inflammation observed in obesity, especially given the gut microbiome's important role in regulating immune function. In obesity, weakened immune responses may both contribute to and worsen gut imbalance, creating a cycle of ongoing inflammation. Adipocytes also show immune-like properties, suggesting that fat cell expansion may represent a response to persistent harmful stimuli. Overall, the microbial hypothesis supports the classical theory by providing a clearer explanation of how microbial factors may trigger and maintain inflammation in obesity [109].

**Figure 3:**
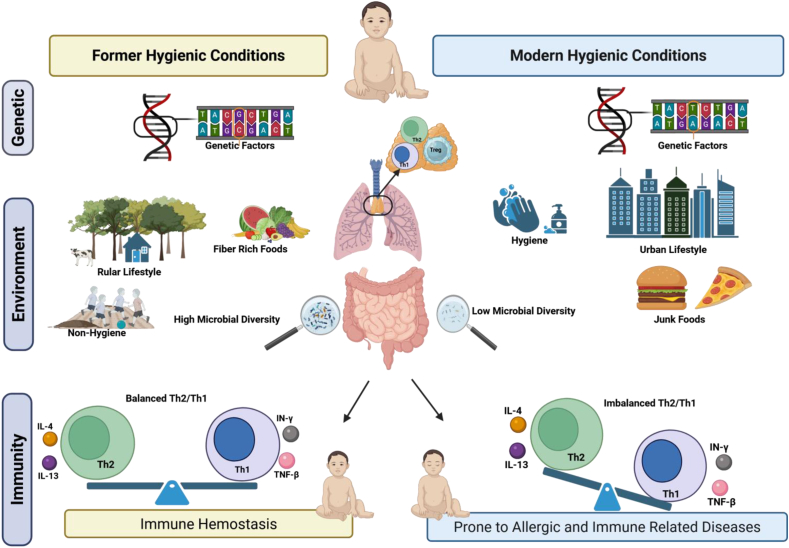
**Genetic, environmental factors, and microbiome shape immunity.** Host genetic variation, shaped by environmental pressures and selection during human evolution, influences immune function. Former hygienic conditions, characterized by rural lifestyles, fiber-rich diets, and high microbial diversity, promote robust regulatory T cell function and a balanced Th2/Th1 ratio, leading to controlled immune responses (immune homeostasis). In contrast, modern hygienic conditions associated with urban living, junk food consumption, and low microbial diversity favor improper Treg activity and Th2 dominance, resulting in chronic inflammation and an elevated risk of developing various diseases, including allergic and immune-related disorders.

In support, several earlier studies have reported that diet and gut microbiota composition act as powerful evolutionary pressures shaping susceptibility to obesity and insulin resistance. Several factors, such as genetic background, dietary patterns, and environment, can shape the human microbiome, which is one of the central regulators of the immune function (Fig. 3). During human evolution, given genes and genetic variants evolved for resistance, particularly against long-term threats such as malaria, smallpox, and tuberculosis, leaving distinct selection patterns shaped by each pathogen's history [95,110]. Dietary components, such as fiber content, can influence the microbiome, gut wall stability, and immunomodulatory biosynthesis [111]. For instance, the soluble fiber-rich diet can increase the frequency of the *Bacteroides fragilis* bacterium, which positively influences the immune system [112]. The resulting bacterial polysaccharide A (PSA) can improve the Th2/Th1 imbalance and the systemic T cell shortage in mice's lymphoid tissues. Moreover, PSA can differentiate CD4+ T cells into Treg cells, which produce IL-10, an essential factor for immune homeostasis and a key regulator of excessive inflammatory responses [113,114]. Along with IR as a potential energy-conservation mechanism for our ancestors, their close contact with diverse microbes could effectively modulate immune responses and prevent chronic inflammation and its unfavorable consequences. Thus, the immune system may have evolved to anticipate and rely on these microbial signals to calibrate the threshold for inflammation.

However, modern lifestyle factors, such as high-fat diets, excessive antibiotic use, and reduced environmental microbial diversity, can disturb this balance, leading to dysbiosis. Dysbiosis refers to a reduction in beneficial bacteria and an overgrowth of harmful species, which can compromise intestinal barrier function and increase gut permeability, a condition commonly known as “leaky gut”. From an evolutionary perspective, intestinal permeability is a functional trade-off between the gut being sufficiently permeable to efficiently absorb nutrients, which was important for survival in ancestral environments with food scarcity, and the need to maintain a sufficient barrier to prevent pathogens' entry and subsequent mucosal immune activation [115,116]. Crucially, this permeability is not simply a passive defect; it is often activated by Na + -dependent nutrient transport to maximize energy yield, a process that optimized caloric maintenance in the past food-scarce environments at the cost of transient barrier reduction [116]. However, this evolutionary adaptation has become maladaptive in the modern environment due to a “mismatch” in nutrient availability and immune signaling. While ancestral barrier opening was likely transient and matched with fiber-fermenting microbiota that rapidly restored integrity, the modern Western diet (constant, hyper-caloric glucose and fat intake) promotes chronic stimulation of these nutrient-dependent opening pathways. Furthermore, the lack of dietary fiber reduces the microbiome's ability to produce short-chain Fatty Acids (SCFAs), such as butyrate, which are crucial for tightening gut junctions and differentiating Treg cells in the gut [117,118]. As a result, the tight junctions, which are open transiently for nutrient uptake, are chronically compromised. This deficiency, combined with the absence of microbial products (e.g. SCFA), fails to provide gut immune homeostasis in the current era.

One key consequence of leaky gut in the context of modern dysbiosis is the translocation of microbial products, particularly LPS, into the bloodstream and the activation of immune system [119]. Once in circulation, LPS binds to LPS-binding protein (LBP), forming a complex that is transferred to CD14, a co-receptor that facilitates its recognition by *TLR4* on immune cells. This interaction activates the NF-κB signaling pathway, leading to the production of pro-inflammatory cytokines such as TNF-α and IL-6. It also drives macrophage polarization toward the M1 phenotype, further amplifying inflammation. In turn, M1 macrophages promote the differentiation of pro-inflammatory T cell subsets, particularly Th1 and Th17 cells, which are central to the development of chronic inflammation. This pro-inflammatory environment is a key driver of insulin resistance [[120], [121], [122]]. Accordingly, the mismatch between immune systems adapted to diverse, fiber-rich, microbially dense ancestral environments and modern Westernized diets, antibiotics, and sanitized lifestyles drives chronic barrier dysfunction, endotoxemia, and low-grade inflammation. This evolutionary perspective not only explains the rising prevalence of metabolic diseases like insulin resistance and type 2 diabetes but also highlights potential interventions, including partially recapitulating ancestral exposures through dietary fiber, prebiotics, and reduced processed foods may restore microbiota balance and mitigate these risks.

Notably, recent research has shown that insulin signaling itself helps maintain gut microbial balance and intestinal barrier integrity, independently of obesity. Lean mice treated with S961, an insulin receptor antagonist, developed insulin resistance alongside early-onset gut dysbiosis, characterized by an overgrowth of pro-inflammatory Proteobacteria, as well as impaired epithelial barrier function due to increased permeability and disrupted cell junctions. Remarkably, discontinuation of S961 treatment rapidly restored both microbial balance and barrier integrity. Fecal transplant experiments further confirmed that the S961-induced dysbiosis directly contributes to barrier dysfunction. These findings highlight insulin signaling as a critical regulator of gut homeostasis and a key defense against microbial imbalance and intestinal inflammation [122]. Therefore, we can hypothesize that a combination of the elevated physical activity, diet regime, and microbiome of early humans might have balanced both the beneficial and detrimental impacts of insulin resistance, potentially leading to a lower risk of chronic diseases in the past. In line with our hypothesis, it has been shown that physical activity can enhance the immune system's ability to fight off infected and cancerous cells. According to an intervention trial, weight reduction resulting from the regular physical activity of obese participants improved natural killer cells and elevated the expression of the corresponding cytokine (IFN-γ) [123]. Therefore, modern immune-metabolic disorders, including allergy, autoimmunity, and insulin resistance, likely arise not from single defects, but from the mismatch between our evolutionarily calibrated physiology and our contemporary environment.

## 6 Population-specific evolutionary adaptations and therapeutic implications

According to population-based studies, there is evidence that susceptibility to insulin resistance and cardiometabolic disease varies across populations, implying complex interactions between genetic architecture, lifestyle, and environmental change. It also reflects ancestral adaptations to regional pathogen burdens and immune responses, in which insulin resistance prioritizes glucose availability to activated immune cells during infection, as we discussed in previous sections. For instance, Pima Indians living in Arizona have one of the highest rates of obesity and insulin resistance worldwide, with approximately half developing type 2 diabetes by mid-adulthood [124]. This population has minimal European admixture and little evidence of type 1 diabetes, resulting in relatively low genetic heterogeneity, which has facilitated the identification of T2D susceptibility loci [124,125]. Additionally, increased expression of pro-inflammatory genes, such as *CCL2* and *CCL3*, has been reported in adipose tissue from obese Pima Indians without type 2 diabetes [126]. Given the established role of pro-inflammatory chemokines (e.g., CCL2, CCL3, CCL4, CCL5, CCL11) in recruiting and activating immune cells during infection, this pattern may reflect an ancestral immune–metabolic program that was advantageous for transient glucose redistribution and host defense during acute infectious stress in our ancestral environment. However, chronic activation of this program in contemporary obesogenic environments becomes maladaptive. Interestingly, Pima individuals living in Mexico exhibit lower insulin resistance even after adjustment for age, sex, and obesity [127], and show markedly lower prevalence of obesity and related metabolic diseases, including type 2 diabetes [127,128]. The prevailing explanation for this contrast could be lifestyle differences, as Mexican Pima maintain a traditional way of life with high levels of physical activity, whereas Arizona Pima follow a more sedentary, Westernized lifestyle.

Additionally, various populations of Pacific Islanders exhibit different insulin resistance and cardiometabolic disease profiles given their lifestyle and gene–environment interaction. Islanders such as the Kitavans, with traditional lifestyle and high-fiber diet, show low prevalence of obesity, T2D, and cardiovascular disease, consistent with preserved insulin sensitivity in this group [129]. In contrast, Pacific Islanders such as Samoan that subjected to rapid nutrition transition, now display the highest rates of obesity and cardiometabolic disease [130], illustrating an evolutionary mismatch between ancestral physiology and modern environment. Interestingly, a missense variant (rs373863828, Arg457Gln) on the *CREBRF* gene is common among Samoans but rare in non-Pacific Island populations. This variant increases BMI by about 1.3–1.5 kg/m^2^ per allele, yet lowers fasting glucose and reduces the risk of T2D at a given BMI. This finding was replicated in other Islanders, including Marianas and Micronesian populations of Guam and Saipan. This study reported the association of minor allele of rs373863828 with HOMA-beta, and likely the higher capacity of insulin secretion that could act against T2D risk in this population [131]. It exemplifies a thrifty genetic variant that was positively selected in the ancestors of these populations to enhance fat storage in adipocytes without clear reduction in insulin sensitivity [132]. A recent study also revealed the co-evolution of immunity and metabolism in Pacific populations. The strong selection signal has been recognized in genes associated with lipid metabolism and immune defense, such as *OSBPL10*, which introgressed from Neanderthals and linked with dyslipidaemia and protection against dengue [133]. This demonstrates an evolutionary trade-off where specific lipid profiles were selected to support robust immune responses. Hence, the high prevalence of dyslipidemia and metabolic diseases in modern Pacific Islanders may represent the maladaptive effect of these ancestral adaptations in the modern environment where patterns of pathogen burdens and food availability have dramatically changed.

Similarly, South Asian populations present a unique evolutionary pattern distinct from other populations, characterized by a ‘thin-fat’ phenotype where type 2 diabetes occurs at lower BMI levels. Compared to other populations, South Asians have lower lean muscle mass, a trait persistent in their ancestors, possibly reflecting an adaptation to climate (thermal stress) or neutral evolutionary event [134], which was hypothesized to account for current T2D susceptibility in this population. Given the prominent role of skeletal muscle in glucose metabolism, lower lean mass can induce long-term peripheral insulin resistance that leads to earlier beta cell exhaustion. This, coupled with deficient beta cell functions and ectopic fat storage in muscle and liver that further reduces insulin action, enhance the risk of T2D among South Asians [135].

For most anti-diabetic drugs, no large, consistent differences in glycaemic efficacy by race/ethnicity have been demonstrated when trials are well-designed and comparable [136]. However, among South and East Asians with early-onset T2D at relatively low BMI and with lower β-cell function, drug classes that induce insulin secretion in response to meal and suppress glucagon (DPP-4 inhibitors, GLP-1 receptor agonists) or blunt post-prandial glucose circulation (α-glucosidase inhibitors) tend to effectively reduce blood glucose levels, compared with European populations [137,138]. Notably, a very recent meta-analysis of GLP-1 receptor agonist in cardiovascular outcome trials demonstrated about twofold greater absolute risk reduction for major adverse cardiovascular events in Asian patients (2.9%) compared to White patients (1.4%) [139]. While factors such as higher relative drug dosage in smaller body sizes may contribute, this difference also aligns with the distinct evolutionary physiology of Asian populations. Specifically, the ancestral “thin-fat” phenotype due to low lean muscle mass, reduced beta-cell function, and a propensity for ectopic fat accumulation in liver and muscle, may render this population particularly responsive to therapies that target lower incretin levels in this population, effectively correcting the specific metabolic vulnerabilities shaped by their evolutionary history.

Moreover, pharmacogenomic studies show that genes involved in drug disposition and action harbor ancestry-structured variation that may contribute to interindividual variability in drug response, though these genetic variants usually explain a small proportion of the variability. For example, rs8192675 in *SLC2A2* gene (coding GLUT2) is associated with greater response to metformin and increased glucose-lowering effect among Europeans. This may be particularly important in African Americans where 49% of the population is homozygous for the effect allele (C), contrary to only 9% in European American [140]. However, a recent genome-wide association study has identified African ancestry-specific variant (rs111770298) associated with reduced metformin response and increased fasting glucose levels in carriers, compared with non-carriers [141]. The proximity of this variant to *FOSL2*, a gene that induce leptin expression in mice, and contains different variants associated with lower triglyceride levels, suggests that ancestral adaptations involving lipid metabolism may pleiotropically influence modern drug responsiveness. This pharmacological evidence serves as a modern clinical assay, revealing how distinct ancestral genetic adaptations, whether related to pathogen defense or famine resilience, may shape differential responses to anti-diabetic therapies in contemporary populations.

## 7 Clinical implications of evolutionary mismatch in the modern era

As discussed, activation of the immune system has critical survival advantages for our ancestors against environmental threats, enabling rapid defense against infections, injuries, and periods of nutrient scarcity. However, these beneficial immune responses become maladaptive in modern environments characterized by over-nutrition, sedentary lifestyle, and persistent low-grade inflammatory stimuli. Importantly, this evolutionary mismatch may explain the diminished protective effects of standard glucose-lowering drugs in patients with T2D against cardiovascular diseases, which do not adequately address the inflammatory processes mediated by trained macrophages [142]. It suggests a need to develop treatments that modulate immune-metabolic crosstalk rather than focusing only on glycemic control. Extending this concept to allergy and immunological disorders further enriches its clinical relevance. The trained immunity and consistent hyper-inflammatory responses of the innate immune system might induce aberrant type 2 inflammation and raise the risk of developing different allergic diseases [143].

Another promising example of evolutionarily informed therapy is developing the Glucagon-like peptide-1 (GLP1) receptor agonist-based therapies, which have revolutionized the treatment of obesity and T2D. GLP1 is an incretin hormone that promotes insulin secretion and suppresses glucagon release, regulating postprandial blood glucose. Also, it reduces appetite, thereby limiting food intake [144]. Interestingly, GLP1 treatment decreases mucus production and airway inflammation, improving obesity-related asthma [145]. However, GLP1 with a very short half-life can act for a limited time (about 1-hour post-meal), which was sufficient for our ancestors who experienced the feast and famine cycles but inadequate for modern humans with persistent access to food resources. Therefore, the short signaling pathway of GLP1 is unable to maintain prolonged satiety, leading to overeating and chronic metabolic disorders. To address this evolutionary limitation, GLP1-based medications such as Semaglutide and Liraglutide with extended half-life and stability were developed [146].

Similarly, the evolutionary role of IgE in immune defensive responses and organismal survival suggests promising potential for applications in cancer immunotherapy. The interaction of IgE with its high-affinity Fc receptors is believed to elicit rapid and potent cytotoxic responses against tumor cells. This mechanism contrasts with traditional therapies that primarily act through suppression or modulation of immune responses. Although IgE-based cancer immunotherapies remain largely in preclinical and experimental phases, existing evidence indicates significant treatment efficacy without inducing type I hypersensitivity reactions, thereby supporting the preliminary safety of this therapeutic approach [147]. This represents a positive example of evolutionary mismatch, whereby an immune mechanism originally adapted to protect our ancestors from pathogens and allergens is now advantageously repurposed for modern cancer treatment. Consequently, understanding the evolutionary base and clinical impact of immune-metabolic mismatch can facilitate the development of medications/interventions for contemporary complex disorders.

## 8 Hypothesis-generating genetics case studies

To illustrate loci where immune and metabolic traits overlap genetically, we examined two regions using public databases, such as Genome-Wide Association Study (GWAS) catalog (https://www.ebi.ac.uk/gwas/), Epigenome-Wide Association Study (EWAS) catalog [148], and population-genetic resources (**see** Supplementary Methods). *PTEN* was selected based on prior hypothesis [90], implicating it in both metabolic regulation and immune signaling, whereas the rs4788084 region (*IL27/NUPR1*) was identified in a data-driven manner through trait associations in public catalogs.

### 8.1 *PTEN* might be evolving under purifying selection in modern humans

**Figure 4:**
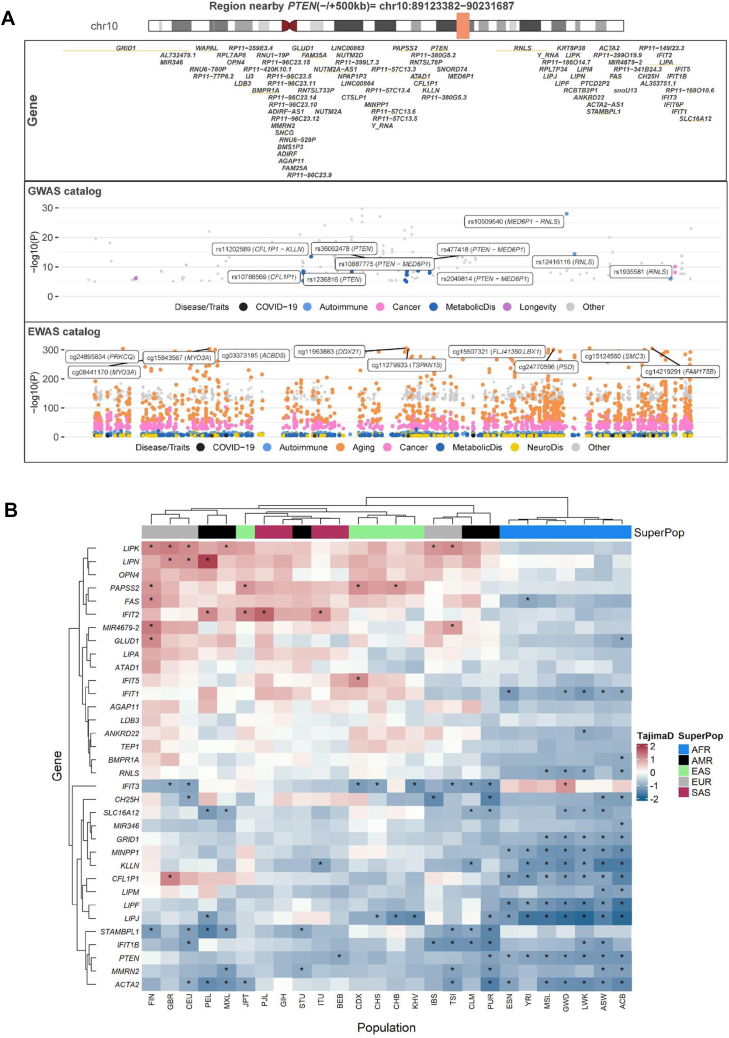
**The *cis*-region (± 500 kb) of *PTEN* is evolving under purifying selection in modern humans. (a)***PTEN* is located on chromosome 10 (89623382–89731687, hg19). The first panel shows the *cis* genes near *PTEN*. The second and third panels show the association with metabolic and autoimmune disorders observed in the GWAS and EWAS catalogs, respectively. **(b)** Distribution of the Tajima's D in 26 human populations of the 1000 Genomes Project (Phase 3). A positive Tajima's D value may reflect population bottlenecks or balancing selection, whereas a negative value may indicate purifying selection, population expansions, selective sweeps, or positive selection.

Earlier research suggested *PTEN* may have “thrifty” properties [90]. This protein also plays a crucial role in the innate immune response by dephosphorylating Ser59 of interferon regulatory factor 3 (IRF3), facilitating its translocation into the nucleus, where it activates the transcription of type I interferon (IFN)-responsive genes. In turn, these activated genes regulate the immune responses mediated by adaptive immune cells [149]. Using GWAS catalog data (hg19), we found that the cis-region (±500 kb) near *PTEN* on chromosome 10 is linked to BMI, waist-hip ratio, T2D, and nearby *MED6P1-RNLS* with type 1 diabetes, COVID-19, and aging (Supplementary Methods). EWAS catalog (hg19) shows altered methylation around *PTEN* associated with T2D, rheumatoid arthritis, and type 1 diabetes, highlighting its role in metabolic and immune disorders (Supplementary Tables 1 and 2, Fig. 4A).

Further to study *PTEN*'s evolutionary pressures, we analyzed 26 populations from the 1000 Genomes Project using the PopHuman database [150] and annotated gene coordinates with org.Hs.eg.db (hg19) in R. We analyzed gene evolution dynamics by calculating Tajima's D value, which detects deviations from neutral evolution by comparing genetic variation patterns. In the African population, all *cis*-genes, except for *LIPN* and *IFIT3*, have Tajima's D value < 0 (Fig. 4B). *PTEN* showed Tajima's D < 0 across all populations, which may indicate an excess of rare alleles, suggestive of purifying selection or recent positive selection [151]. *PTEN* clustered with *ACTA2, MMRN2, STAMBLP1,* and *IFIT1B*, hinting at possible functional co-evolution. Notably, *IFIT1B* belongs to the interferon-induced protein family, linking immunity and metabolism. Given PTEN's role as a negative regulator of PI3K/AKT signaling [152], central to insulin sensitivity and immune cell function [153], low Tajima's D in this region may suggest minor genetic changes may impact these pathways.

### 8.2 Distinct evolutionary patterns surrounding *IL27* and *NUPR1* across populations

**Figure 5:**
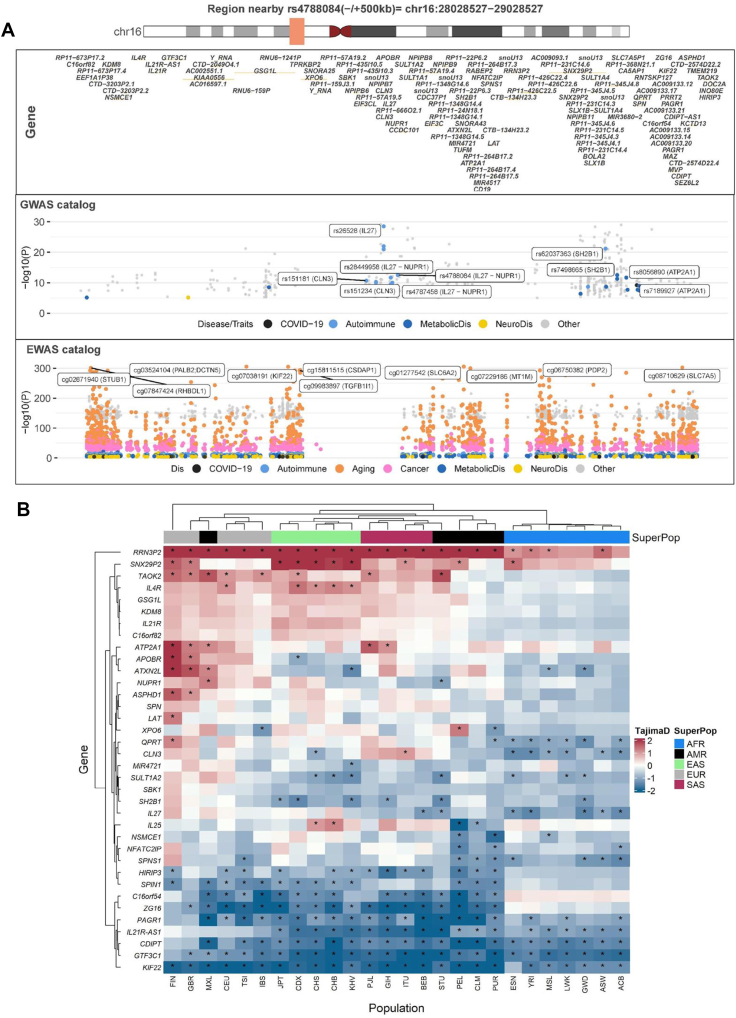
**Distinct evolutionary patterns in the *cis*-regions (± 500 kb) of *IL27* and *NUPR1* across populations. (a)** The *cis*-region (±500 Kb) of rs4788084 is associated with obesity-related traits, such as BMI, waist-hip ratio, body fat percentage, and autoimmune disorders, including type 1 diabetes, Crohn's disease, and inflammatory bowel disease. rs4788084 is located on chromosome 16 (28539848 C > T, hg19). The first panel shows the *cis* genes near rs4788084. The second and third panels illustrate the association with metabolic and autoimmune disorders observed in the GWAS and EWAS catalogs, respectively. **(b).** Distribution of the Tajima's D value in 26 human populations of the 1000 Genome Project (Phase 3). A positive Tajima's D value may reflect population bottlenecks or balancing selection, whereas a negative Tajima's D value may indicate purifying selection, selective sweeps, or positive selection.

Using Open Targets Genetics (https://genetics.opentargets.org/) and the GWAS catalog (Supplementary methods), we identified a cis-region (±500 Kb) near rs4788084 linked to BMI, waist-hip ratio, body fat percentage, type 1 diabetes, Crohn's disease, and inflammatory bowel disease (Supplementary Tables 3 and 4). Given that BMI, waist circumference, waist-hip ratio, and body fat percentage are key obesity measures [154], we examined genes near rs4788084, focusing on *IL27* and *NUPR1*. IL-27, a cytokine in the IL-6/IL-12 family, improves insulin resistance, enhances thermogenesis, and reduces diet-induced obesity through IL-27 receptor signaling [155]. IL-27 also contributes to the pathogenesis of T1D. NUPR1 is crucial for protecting β-cells from apoptosis, preventing the degradation of insulin stores, and ensuring proper insulin secretion during inflammatory and obesity-related tissue stress [156]. Thus, we further explored how DNA methylation might influence this *cis*-region (±500 Kb) (Fig. 5A and Supplementary Table 5) and found significant association with T2D. Additionally, evolutionary trajectories and selective pressures near rs4788084 across 26 human populations reveal distinct evolutionary patterns for *IL27* and *NUPR1* genes (Fig. 5B). Both genes show negative Tajima's D values in African and South Asian populations, suggesting recent selective sweeps or purifying selection. *NUPR1* consistently shows positive Tajima's D values across European groups, suggesting balancing selection. This could indicate that multiple alleles of *NUPR1* are being maintained in these populations, possibly due to heterozygote advantage or fluctuating selection pressures. *IL27*, on the other hand, only shows positive Tajima's D in Finland (FIN) and CEU (Utah Residents with Northern and Western European Ancestry) populations. This localized positive selection might reflect specific immune challenges or environmental factors unique to these groups. Selection acting on *IL27* and *NUPR1* in this population may also likely reflect evolutionary pressures to balance immune defense and tissue protection during infection, rather than simply reducing inflammation or insulin resistance at all times.

Together, these loci are presented as descriptive, hypothesis-generating examples of genomic regions where immune- and metabolic–trait associations co-localize in public datasets. The analyses are intended to document patterns of trait overlap and population-genetic variation rather than to infer causality or adaptive significance. Further functional or fine-mapping studies would be required to determine the biological relevance of specific variants.

## 9 Limitations and future perspectives

Exploring obesity-related IR and immunity through the lens of evolutionary medicine presents both significant challenges and opportunities. One of the main challenges is understanding how genetic, environmental, and lifestyle factors interact to influence IR. Evolutionary medicine often uses broad historical perspectives, which can make it difficult to establish direct links between past evolutionary pressures and current health issues. Today's environment, characterized by an abundance of food and sedentary lifestyles, is vastly different from the conditions our ancestors faced. This discrepancy can limit the applicability of evolutionary theories, as the selective pressures that shaped our ancestors are not the same as those we experience now. However, studying IR from an evolutionary perspective can shed light on why certain traits that were once beneficial might now contribute to health problems like obesity and related metabolic disorders. For example, insulin resistance likely conferred a survival advantage during periods of famine by prioritizing glucose supply to the brain and immune system, and during infection by supporting effective immune responses. These adaptations, while beneficial in ancestral environments, can become maladaptive in today's context of chronic caloric abundance and reduced pathogen exposure [21,157,158]. Bridging this evolutionary insight with modern biomedical research is essential for understanding the full complexity of metabolic diseases and for identifying effective points of intervention. To address these challenges, integrating advanced technologies such as multi-omics, machine learning, and genome editing approaches on model organisms offers promising research and intervention opportunities.

Model organisms such as mice and zebrafish are essential tools for investigating how both ancestral and modern environments influence physiology. Under controlled conditions, they can be exposed to environmental stressors like starvation, infection, or nutrient excess, allowing researchers to test evolutionary hypotheses related to insulin resistance and immune function. Comparative and phylogenetic studies using these models help uncover conserved and divergent metabolic and immune pathways shaped by specific ecological pressures. By observing how these organisms respond to different challenges, scientists can better understand the evolutionary forces that have shaped human biology. This approach not only clarifies the interaction between adaptive traits and current health issues but also informs strategies for addressing obesity-related insulin resistance in modern populations. Using the phylogenetic approach, earlier, we also found that T2D-related genes are evolving under purifying selection in Drosophila [159]. This indicates that deleterious mutations in these genes are selectively removed to preserve their crucial roles in processes such as insulin signaling, glucose homeostasis, and energy metabolism, which are highly conserved and fundamental for organismal survival [21,159]. However, the amino acid residues of Arg273 and Thr323 in CG805, Thr277 in *ZnT35C*, and Ala499 in kar protein exhibited positive selection, suggesting adaptive evolution in response to environmental changes [159]. This information promises valuable insights for evolutionary medicine, which could result in effective biomarker and drug discovery.

## 10 Conclusion

Insulin resistance, though implicated in a spectrum of modern metabolic and inflammatory disorders, appears to be an evolutionarily conserved mechanism that once conferred critical survival benefits. By reallocating glucose to insulin-independent tissues such as the brain and immune system, IR may have supported host defense and cognitive function during periods of infection or scarcity. In contemporary environments, however, marked by caloric excess and physical inactivity, this adaptive trait has become maladaptive, causing chronic inflammation and disease. Genomic signals of purifying selection near genes such as *PTEN*, *IL27*, and *NUPR1* underscore the evolutionary importance of the IR-immune axis. Understanding insulin resistance as an evolutionary mismatch reframes it not simply as pathology, but as a vestige of past resilience-one that may hold keys to addressing today's global burden of metabolic disease.

## CRediT authorship contribution statement

**Maryam Moazzam-Jazi:** Writing – review & editing, Writing – original draft, Visualization, Software, Resources, Project administration, Methodology, Investigation, Formal analysis, Data curation, Conceptualization. **Saeideh Jafarinejad-Farsangi:** Writing – original draft. **Leila Najd-Hassan-Bonab:** Data curation. **Maryam Daneshpour:** Writing – review & editing. **Zhaoli Liu:** Writing – review & editing. **Manoj Kumar Gupta:** Writing – review & editing, Writing – original draft, Visualization, Validation, Supervision, Software, Resources, Project administration, Methodology, Investigation, Formal analysis, Data curation, Conceptualization. **Ramakrishna Vadde:** Writing – review & editing, Supervision.

## Ethics approval and consent to participate

Not applicable. This study involves only computational analyses of publicly available data and does not include any studies with human participants or animals.

## Consent for publication

Not applicable.

## Funding

None.

## Declaration of competing interest

The authors declare that they have no known competing financial interests or personal relationships that could have appeared to influence the work reported in this paper.

## Data Availability

No data was used for the research described in the article.
