# Supplementary material for: Interplay between obesity-associated insulin resistance and immune system through the lens of evolutionary medicine
Source: Mol Metab. 2026 Feb 13;106:102335. doi: 10.1016/j.molmet.2026.102335 (PMC12954304; doi:10.1016/j.molmet.2026.102335)
Supplement: Multimedia component 2 [file mmc2.docx]

**Supplementary Methods: Population-Genetic Analyses**

**Trait prioritization**

To explore how natural selection shaped immune-metabolic gene signatures across human populations, we first examined genetic associations at the PTEN locus with T2D and immune traits using public GWAS/EWAS data. *PTEN* was prioritized based on earlier studies proposing it as a putative thrifty gene for metabolic disease risk in modern environments [1]. We then identified the rs4788084 locus (*IL27/NUPR1* region) through shared GWAS signals for metabolic and immune traits for evolutionary analyses.

**Data Sources and Variant Selection**

To investigate *PTEN* function, we initially examined the cis-region region nearby its (chr10:89123382-90231687) cis-region (±500 kb) using genome-wide association studies (GWAS) and epigenetic-wide association studies (EWAS) data (hg19) from the gwascat package [2] in R (**Supplementary Tables 1** and **2**) . While investigating potential variants linked to both obesity-related traits and autoimmune disorders using the Open Targets Genetics (https://genetics.opentargets.org/) and GWAS catalog (<https://www.ebi.ac.uk/gwas/> ). We also identified a *cis*-region (± 500 Kb) near rs4788084 (chr16:280228527-29028527) associated with BMI, waist-hip ratio, body fat percentage, type 1 diabetes, Crohn’s disease, and inflammatory bowel disease (**Supplementary Tables 3** and **4**).

**Population-Genetic Statistics**

Further, to investigate the evolutionary trajectories and selective pressures on *PTEN* across 26 human populations from the 1000 Genomes Project (PHASE3) (<https://www.internationalgenome.org/>), we used the PopHuman database [3]. Gene coordinates were annotated using the [org.Hs.eg.db](https://bioconductor.org/packages/release/data/annotation/html/org.Hs.eg.db.html) package [4] (hg19) in R. We analyzed gene evolution dynamics by calculating Tajima’s D value, which detects deviations from neutral evolution by comparing genetic variation patterns. Tajima's D = 0 indicates neutral evolution. Tajima's D < 0 (negative values) signals excess rare alleles consistent with recent population expansions, selective sweeps, or purifying selection removing deleterious variants or positive selection; Tajima's D > 0 (positive values) reflects excess intermediate-frequency alleles typically from balancing selection maintaining polymorphisms or population bottlenecks reducing diversity [5, 6].

**Limitations**

Tajima's D are summary statistics confounded by demography, recombination, and mutation rate heterogeneity [7] . No causality is inferred; findings are **hypothesis-generating** requiring functional validation. All data are publicly available.

**References**

1. Venniyoor A. PTEN: A Thrifty Gene That Causes Disease in Times of Plenty? Frontiers in Nutrition. 2020;7. doi: 10.3389/fnut.2020.00081.

2. Carey V. gwascat: Representing and Modeling Data in the EMBL-EBI GWAS Catalog. 2024.

3. Casillas S, Mulet R, Villegas-Mirón P, Hervas S, Sanz E, Velasco D, et al. PopHuman: the human population genomics browser. Nucleic Acids Research. 2017;46(D1):D1003–D10. doi: 10.1093/nar/gkx943.

4. Carlson M, Falcon S, Pages H, Li N. org. Hs. eg. db: Genome wide annotation for Human. R package version. 2019;3(2):3.

5. Gupta A, Dey CS. PTEN, a widely known negative regulator of insulin/PI3K signaling, positively regulates neuronal insulin resistance. Mol Biol Cell. 2012;23(19):3882–98. doi: 10.1091/mbc.E12-05-0337.

6. Carlson CS, Thomas DJ, Eberle MA, Swanson JE, Livingston RJ, Rieder MJ, et al. Genomic regions exhibiting positive selection identified from dense genotype data. Genome Research. 2005;15(11):1553–65. doi: 10.1101/gr.4326505.

7. Jensen JD, Kim Y, DuMont VB, Aquadro CF, Bustamante CD. Distinguishing Between Selective Sweeps and Demography Using DNA Polymorphism Data. Genetics. 2005;170(3):1401–10. doi: 10.1534/genetics.104.038224.
